# The Effect of Different Attentional Focus Instructions during Finger Movement Tasks in Healthy Subjects: An Exploratory Study

**DOI:** 10.1155/2017/2946465

**Published:** 2017-02-14

**Authors:** Giacomo Rossettini, Marco Testa, Marco Vicentini, Paolo Manganotti

**Affiliations:** ^1^Department of Neuroscience, Rehabilitation, Ophthalmology, Genetics, Maternal and Child Health, University of Genova, Campus of Savona, Via Magliotto 2, 17100 Savona, Italy; ^2^Psychiatric Hospital “Villa Santa Chiara”, Verona, Italy; ^3^Clinical Neurology Unit, Department of Medical Science, Surgery and Health, University of Trieste, Via Farneto 3, 34142 Trieste, Italy

## Abstract

External focus of attention (EFA) and internal focus of attention (IFA) represent commonly used strategies to instruct subjects during exercise. Several studies showed EFA to be more effective than IFA to improve motor performance and learning. To date the role of these strategies on motor performance during finger movement was less studied. The objective of the study was to investigate motor performance, patient's preference induced by IFA and EFA, and the focus during control condition. Ten healthy right-handed participants performed a finger movement task in control, EFA, and IFA conditions (counterbalanced). Errors, patient's preference, and type of attentional focus spontaneously adopted during the control condition were recorded. EFA determined less error (*p* < 0.01) compared to control and IFA. Participants preferred EFA against IFA and control condition. In the control group 10% of subjects adopted a purely EFA, 70% of subjects adopted a purely IFA, and 20% of subjects adopted a mixture of the two foci. Our results confirm that EFA is more effective than IFA and control in finger movement task. Due its clinical relevance, the interaction between attention and finger movement should be further investigated.

## 1. Introduction

The achievement of an effective and stable motor performance represents an everyday challenge in sport, exercise, and rehabilitation setting [1]. These goals are pursued by different cognitive facilitations such as verbal instructions that recall the attentional focus of performer to specific elements [2]. The internal focus of attention (IFA) is directed to the specific body segments involved in producing a movement (e.g., a limb), while the external focus of attention (EFA) is directed to a specific outcome or to the effects produced by the movement on the environment (e.g., a target, the implement, or apparatus) [3]. Researchers reported that EFA, more than IFA, influences motor performance and effective learning in healthy subjects [3, 4] and in patients with musculoskeletal disorders and central nervous system diseases [5–7]. The benefit of EFA has been shown in movement efficacy, efficiency, and kinematics and extends across different types of tasks, skill levels, and age groups [3].

To explain these findings the* Constrained Action Hypothesis* was proposed [8]. According to this theory IFA constrains the motor system and worsens the quality of motor execution by requiring a higher level of conscious control; instead EFA promotes automatic, unconscious, fast, and reflexive control processes, which underpin the effectiveness (e.g., accuracy, consistency, and reliability in achieving the goal) and efficiency (e.g., fluent and economical executions and automaticity) of motor performance [9, 10]. Some evidence seems to confirm the higher level of efficiency obtained in EFA condition as it was associated with a reduced cognitive demand during the execution of the task [8], an increased low amplitude and high frequency of movement adjustments [9–11], a reduced electromyography activity [12–16], a greater movement fluidity [17], and a reduction of oxygen consumption [18]. Moreover the role of attention in motor control is renown [19] and numerous studies conducted on the field have examined the effect of attentional focus on accuracy outcome measures. Examples of tasks examined were balance [11, 20], force production [12, 13], basketball free throw shot [14], golf swing [21], single leg movement [17], long jump [22], gymnastic [23], sprint [24], and physical performance [25].

Despite the wide interest in this area of research, several aspects have received less consideration. The first aspect is about the effect of attentional focus on fine motor skill such as fingers movement [26]. Although Duke et al. [27] provided initial evidence that EFA compared to IFA is more effective to determine an improvement of the accuracy of playing a passage at the music keyboard, however generalizability of these findings remains questionable. Indeed, they investigated without a control condition and in absence of attentional instruction a population of expert musicians. The second aspect concerns the “manipulation check” of the participant's attentional focus spontaneously adopted in neutral condition that should be known to avoid bias of results [28]. For instance Ille et al. [29], during a sprint start performance, found that most participants adopted an IFA instruction. At opposite, other authors in a running task showed that the predominant focus was EFA [30, 31]. Instead, Porter et al. [32] revealed that participants used predominantly a mixed focus of attention during an agility task. This heterogeneity of attentional strategy observed in neutral condition seems not to be related to a specific typology of motor task or subjects' expertise but represents a part of the natural learning process [33, 34] in which participants focus on movement, environment, or a combination of these elements in search of the most efficient motor program [32]. The third is the assessment of individual preference of attentional focus that can be considered as motivational factor capable of interacting with motor performance [35]. Individual preferences seem to play a role in attentional research [36, 37], but findings are still controversial. One set of studies reported that participants preferred the IFA instructions to the EFA during motor performance of force production [38] and basketball task [39]. Others report EFA as the preferred attentional strategy during balance [10] and dart throwing [40, 41].

To our knowledge, the effect of attentional focus strategies during finger movement task, the analysis of focus during the neutral condition, and of the participant's preference represent few aspects still to be investigated. In this exploratory research we aimed to investigate the performance obtained by healthy subjects at a finger's motor task, under instructed EFA and IFA and in control conditions without specific instructions about the focus of attention. Additionally, we assessed what subjects were focusing on during the control condition and which condition the subjects preferred. We expect that subjects

1. in EFA condition achieve a better motor performance compared to IFA and control;
2. in control condition spontaneously adopt more IFA strategy;
3. perceive EFA strategy as more effective.

## 2. Methods

### 2.1. Participants

Participants were 10 healthy right-hand-dominant students from Verona University, aged 20–32 years (M age = 28.1 years; SD = 2.64; 4 women, 6 man) as assessed by the Edinburgh Handedness Inventory [42]. All subjects were recruited with convenience sampling. They were screened to ensure that they had no previous expertise in playing any music instruments. They were naïve to the purpose and the task of the research. No participants either received course credit or money for their participation. The Institutional Review Board of Verona University approved the research and written informed consent was obtained from participants. The experiment was undertaken in accordance with the Code of Ethics of the World Medical Association (Declaration of Helsinki).

### 2.2. Task and Instrumentation

The research was conducted in a quiet room. Subjects were seated in a comfortable chair with elbows semiflexed and forearms pronated, fully relaxed, and supported by the arm of the chair. A computer keyboard (Apple MacBook 13.3), positioned at a distance of 60 cm on a support placed in front of the subject, was used for the execution of the task. The QWERTY keyboard was masked with a plastic cover leaving free only 5 keys (F, T, Y, U, and K). The required task was to press by the all five fingers of the right hand 20 times on the keyboard's keys respecting the following sequence: thumb, index, middle finger, annular, little finger; thumb, middle finger, little finger, index, annular; little finger, annular, middle finger, index, thumb; little finger, middle finger, thumb, annular, index. Each finger was paired with a single key and the contact between finger and key had to be maintained during the whole tasks. A digital metronome program (Metronomo, Version 1.1 Robert Wessels Dutch:Apps) guided the sequential execution of the fingers' movement. The 1 hz frequency of the metronome determined the speed of execution.

### 2.3. Experimental Design and Procedure

We employed a within-subject design in which all subjects performed in three conditions (control, IFA, and EFA). The trials were performed in the morning (h. 9-10) in one day. Subjects were instructed to take only a light meal with no caffeine at least 2 h before testing. For all the conditions a limited number of trials was chosen in order to avoid fatigue effects [29].

A familiarization phase was completed only in control condition [25, 32]. At first, participants received a detailed explanation and demonstration of the task by the investigator. All subjects improved the motor task without attentional instructions (control condition). During the control condition subjects were instructed as follows: “from now on, please perform the exercise.” After 5 min of free training, each participant executed 1 block of 7 tasks as the control trial. A 5-seconds pause was given between any tasks. Later on, subjects performed the trials with verbally instructed attentional focus. The order of the IFA or EFA condition was counterbalanced for all participants to control for order effect [25, 32]. Subjects performed 1 block of 7 tasks for any attentive condition (EFA, IFA). We gave a 5-second pause between any task and 5-minute pause between blocks. The attentional conditions were defined by verbal instructions provided to subjects before starting each task as follows: “from now on, please focus on the target keys” (EFA condition); “from now on, please focus on your moving fingers” (IFA condition).

**Figure 1:**
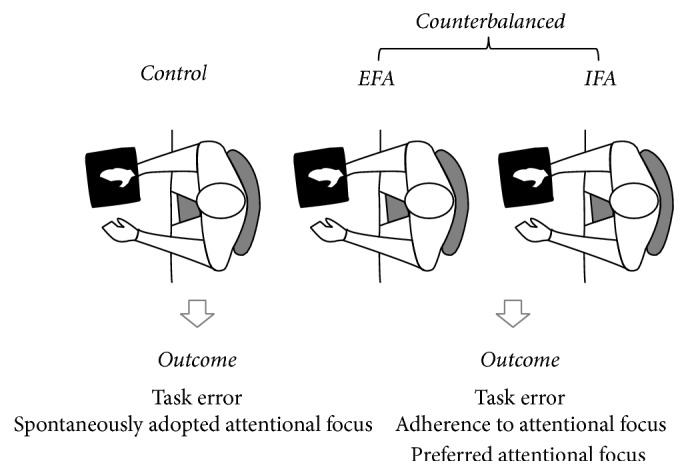
Experimental procedure. Note that participants performed a motor task with right hand under different attentional conditions. They started without any attentive instruction (control). Subsequently they executed the task under external focus of attention/internal focus of attention in a counterbalanced way. EFA is external focus of attention; IFA is internal focus of attention.

No guidelines, encouragement, and verbal or visual feedback were provided to participants and the researcher was the only individual present with the participant during all trials to control for the influence of social factors [12]. A graphical representation of the experimental procedure is presented in Figure 1.

### 2.4. Behavioural Outcome

A text file was used to record and check the task errors (TE) (e.g., omitted key typing, wrong typing order) of the task. The total number of errors for each experimental condition was used to assess the difference of accuracy performance between attentional conditions.

### 2.5. Subjective Outcome

Participants were questioned in order to determine which condition they preferred and which one they considered more effective for the execution of the task [10]. The subjects' response was used to evaluate their preferred attentional focus among IFA, EFA, and control.

### 2.6. Manipulation Check

After the execution of the control condition trial, the experimenter interviewed the participants to know the type of attentional focus that they had adopted [29]. Two authors coded participants' answers into three categories. Items relating to the goal of the task (i.e., the keys, the sequence to be pushed) were coded as EFA. Items related to body parts or to the movement (i.e., the finger motion) were coded as IFA. The “other” category comprises items related to both IFA and EFA condition or to an attentional focus that was considered neither external nor internal (e.g., thinking to nothing) [32].

Moreover, at the end of the experiment, participants have to indicate how much (%) of the time they were able to follow the indicated IFA and EFA condition [18].

### 2.7. Statistical Analysis

By *R* Statistical Environment, task error (TE) was assessed by one way, repeated measures ANOVA, with “condition” as within group factor and three levels (EFA, IFA, and control). Descriptive analysis was used to assess subjects' preference of focus of attention strategy and spontaneous choice of strategy at the control condition. Data are shown as mean ± standard deviation (SD) in the text and tables and standard error (SE) in figures. Statistical significance was set as *p* < 0.01.

## 3. Results

### 3.1. Motor Performance Data

**Figure 2:**
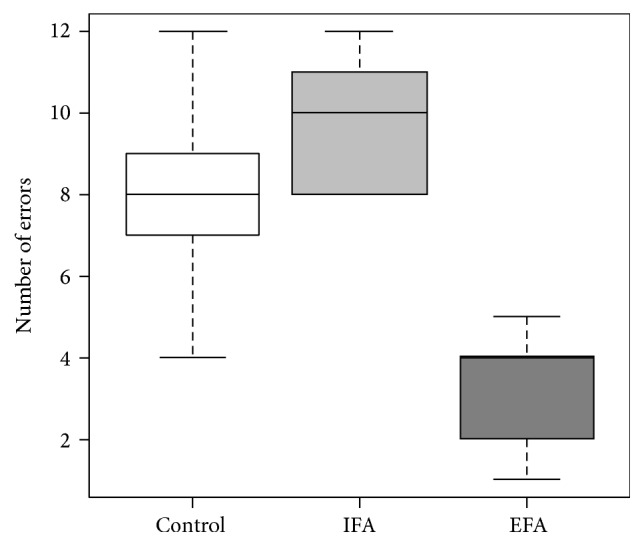
Outcome: motor performance data. Note that the plot displays the motor performance outcomes under different attentional conditions. External focus of attention exhibits a significant lower number of errors (*p* < 0.01). Not significant differences were found between control and internal focus conditions. EFA is external focus of attention; IFA is internal focus of attention.

As far as we are concerned with the total number of task error (TE) we observed a significant lower number of errors (*F*_2,27_ = 37.01, *W*_2_ = 20.05, *p* < 0.001) between EFA instructed condition (M = 3.4, SD = 1.35), control condition (M = 8.2, SD = 2.20), and IFA instructed condition (M = 9.9, SD = 1.60) (Figure 2). No differences were found between control and IFA (Tukey HSD multiple comparison test of means *p* < 0.10).

### 3.2. Preference towards a Specific Attentional Focus Conditions

At last, when asked for their preferences, subjects reported as preferred focus the EFA condition (60%) against IFA (20%) and control (20%) conditions.

### 3.3. Attentional Focus under the Neutral, Control Condition

10% of subjects adopted a purely EFA, 70% of subjects adopted a purely IFA, and 20% of subjects adopted a mixture of the two. A detailed explanation of subject's focus adopted during control condition was described in Table 1 (reported using different foci on different tasks, or some balance of internal and external focus across tasks).

### 3.4. Ability to Follow the Indicated Attentional Condition

For the IFA condition, subjects indicated compliance 90.4% (sd = 4.8%) of the time, for EFA 84.1% (sd = 5.2%).

## 4. Discussion

### 4.1. Main Findings

The purposes of the current study were to investigate the effect of specific attentional instructions on motor performance during a finger movement task, the participants' subjective preference of the focus of attention strategy, and the subject's attentional focus adopted under the neutral control condition. The main finding was that verbally instructed EFA condition produces better performance as reduced number of errors during a finger movement than IFA and control. Our results confirm and expand the observation of Duke and colleagues detected in a sample of expert musicians [27] and corroborates the existing literature on attentional focus in healthy subjects [3, 4].

If we refer to the* Constrained Action Hypothesis* [8–10] and the more recent* OPTIMAL theory* [35], we can speculate that EFA helps motor system during selection and execution of motor response facilitating functional connectivity across brain regions. Indeed EFA, emphasizing the tactile information derived from finger-key contact, increases the input to somatosensory areas closely connected to motor areas and sustains an effective neural connections in support of motor performance [26]. Instead, IFA operates as a self-invoking trigger that worsens the motor performance by focusing on the proprioceptive information from the finger [43]. Both these submodalities of the haptic sense are involved in motor planning and control of the effector [28], but the exteroceptive source (in our case the tactile sensation derived from the keys) seems to be the more relevant for a fine motor control of the fingers [44–46]. Furthermore, no differences were found between the motor performance obtained in IFA and control conditions. This observation is in line with the previous research on attentional focus [11, 20, 22–25, 32] and suggests that subjects without a specific focus might spontaneously direct their attention to the body segment's movement, disrupting automatic processing in a manner similar to those who were instructed to adopt an IFA strategy [22].

Our “manipulation check” provides direct support to this assumption. Indeed 70% of the subjects in control group spontaneously used a pure IFA strategy. Although our results are congruent with the observation of Ille et al. [29], the literature is still conflicting on this topic. Schücker et al. [31] suggested that the predominant focus during control condition was EFA in the majority of analysed subjects (*n* = 15/20), or along most (64.38%) of the exercise time [30], whereas Porter et al. [32] revealed that participants used a mixed focus of attention in about 77% of the trials. Despite the spontaneous choice taken in control condition, the subjects, after experiencing the different strategies, declared a strong preference for EFA compared to other conditions (IFA and control). Our results contribute to the reinforcement of the evidences that support preferentiality for EFA strategy with respect to the IFA [10, 40, 41] as a cognitive tool capable of facilitating the motor performance.

Nevertheless, coaches and physiotherapist by far prefer and use IFA strategy during their sessions of sport training and rehabilitation. In a recent analysis emerged that patients after stroke are frequently encouraged to direct their attention more to body movement (IFA) than movement effects [47, 48]. Similarly, elite athletes habitually received more instructions related to body and limb movement rather than oriented to the goal of task [49, 50]. This approach could reduce automaticity and performers' opportunity to demonstrate what they can achieve themselves, hindering performance and learning [21, 51].

The participants' preference for a specific attentional focus should be attentively considered when setting up a motor task since it might influence performance and learning in a positive (preference towards EFA) or negative way (preference towards IFA) [52]. From a practical perspective, whenever it is possible, coaches and physiotherapists should give to the subjects the possibility of experiencing the different results obtained by the two attentional strategies. They should assess the individual preference [5], provide goal-oriented instructions, and reinforce the adoption of EFA, enhancing expectancies and supporting autonomy [53]. These strategies, by strengthening the coupling of goals to actions, can increase the motivation and interest to the task and create the optimal condition for motor performance execution [35].

### 4.2. Limits

Elevated task difficulty is necessary to observe a beneficial effect of attentional instructions [20]. As the estimation of a task complexity is very difficult [54], in this experiment, we adopted a finger movement task composed by 20 pressures that we considered of adequate level of difficulty, according to the methodology adopted by Duke et al. [27] who proposed a task with 16 fingers movements. Verbal instructions can be misleading and introduce confounds, but we tried to avoid these biases keeping the instructions simple. We were very careful setting up the instructions of which structure was standardized and differed only by few words [3] when directed to the goal (EFA; keys) or to the movement (IFA; fingers). Their correct comprehension was confirmed by the results of our “manipulation check” which showed that all participants were able to follow the indicated attentional instruction. Additionally, in this study we used a within-subject design with a control group. Potential bias effects from separate groups differences due to interindividual variances were minimized by adopting a within subjects design of the study [24]. The control condition was proposed first [25, 32] in order to avoid the potential influence of previous IFA/EFA instructions on the spontaneity of motor strategy choice. We cannot exclude that this solution could have induced a “practice effect” on the following IFA/EFA conditions, reducing the comparability of the performance obtained in control condition with that obtained in IFA/EFA conditions. Small sample, the lack of a priori sample size calculation, the analyses of a single motor performance recorded after acquisition phase, the lack of kinematic and neurophysiologic data, the use of descriptive analysis for subject's preference, and spontaneous choice of strategy at the control condition limit the weight of the present study. Future researches should use a wider sample and implement neurophysiological outcome measures [26] to investigate the effect of learning attentional focus during finger motion task considering also retention and transfer phase [3].

## 5. Conclusion

In summary, the EFA strategy influence positively the motor performance more than IFA and control and is preferred by the subjects. This exploratory study confirms and expands previous research on this field. Moreover it may help in designing future experimental trial regarding the role of attentional focus applied to fine finger movement tasks in patients with musculoskeletal dysfunction and neurological disorders.

## Figures and Tables

**Table 1:** Attentional focus under the neutral, control condition. Note that the table reports a detailed explanation of attentional focus adopted by subjects during the neutral, control condition. EFA is external focus of attention; IFA is internal focus of attention.

Subject	“What's your focus during the control condition?”	Code
(1)	“…I was thinking to my fingers…”	IFA
(2)	“…Sometimes I focused to my fingers, sometimes to the sequence of keys…”	IFA/EFA
(3)	“…I was thinking to the motion of my fingers…”	IFA
(4)	“…I reflected to my hand…”	IFA
(5)	“…I started to focus on the movement of my fingers, but afterwards I focused to the keys…”	IFA/EFA
(6)	“…I considered the motion of my fingers…”	IFA
(7)	“…I was focusing to my fingers…”	IFA
(8)	“…I was thinking to the sequence of keys…”	EFA
(9)	“…The movement of the fingers captures my attention…”	IFA
(10)	“…I was focusing to the motion of my fingers…”	IFA
